# Characterisation of a novel [^18^F]FDG brain PET database and combination with a second database for optimising detection of focal abnormalities, using focal cortical dysplasia as an example

**DOI:** 10.1186/s13550-023-01023-z

**Published:** 2023-11-15

**Authors:** Sameer Omer Jin, Inés Mérida, Ioannis Stavropoulos, Robert D. C. Elwes, Tanya Lam, Eric Guedj, Nadine Girard, Nicolas Costes, Alexander Hammers

**Affiliations:** 1 Faculty of Life Sciences and Medicine, School of Biomedical Engineering and Imaging Sciences, King’s College London, London, UK; 2 Centre d’Etude et de Recherche Multimodale et Pluridisciplinaire en Imagerie du Vivant (CERMEP), Lyon, France; 3 Department of Clinical Neurophysiology, King’s College Hospital, London, UK; 4 Department of Basic and Clinical Neuroscience, Institute of Psychiatry, Psychology, and Neuroscience, King’s College London, London, UK; 5 Children’s Neuroscience Centre, Evelina London Children’s Hospital, Guy’s and St Thomas’ NHS Trust, London, UK; 6 Nuclear Medicine Department, APHM, CNRS, Centrale Marseille, Institut Fresnel, Timone Hospital, CERIMED, Aix Marseille University, Marseille, France; 7 Department of Neuroradiology, APHM, CRMBM, UMR CNRS 7339, Timone Hospital, Aix Marseille University, Marseille, France; 8 King’s College London & Guy’s and St Thomas’ PET Centre, London, UK

**Keywords:** Statistical parametric mapping (SPM), Harmonisation, Anomaly detection, Interscanner differences, Smoothness

## Abstract

**Background:**

Brain [^18^F]FDG PET is used clinically mainly in the presurgical evaluation for epilepsy surgery and in the differential diagnosis of neurodegenerative disorders. While scans are usually interpreted visually on an individual basis, comparison against normative cohorts allows statistical assessment of abnormalities and potentially higher sensitivity for detecting abnormalities. Little work has been done on out-of-sample databases (acquired differently to the patient data). Combination of different databases would potentially allow better power and discrimination. We fully characterised an unpublished healthy control brain [^18^F]FDG PET database (Marseille, *n* = 60, ages 21–78 years) and compared it to another publicly available database (MRXFDG, *n* = 37, ages 23–65 years). We measured and then harmonised spatial resolution and global values. A collection of patient scans (*n* = 34, 13–48 years) with histologically confirmed focal cortical dysplasias (FCDs) obtained on three generations of scanners was used to estimate abnormality detection rates using standard software (statistical parametric mapping, SPM12).

**Results:**

Regional SUVs showed similar patterns, but global values and resolutions were different as expected. Detection rates for the FCDs were 50% for comparison with the Marseille database and 53% for MRXFDG. Simply combining both databases worsened the detection rate to 41%. After harmonisation of spatial resolution, using a full factorial design matrix to accommodate global differences, and leaving out controls older than 60 years, we achieved detection rates of up to 71% for both databases combined. Detection rates were similar across the three scanner types used for patients, and high for patients whose MRI had been normal (*n* = 10/11).

**Conclusions:**

As expected, global and regional data characteristics are database specific. However, our work shows the value of increasing database size and suggests ways in which database differences can be overcome. This may inform analysis via traditional statistics or machine learning, and clinical implementation.

**Supplementary Information:**

The online version contains supplementary material available at 10.1186/s13550-023-01023-z.

## Introduction

Neuroimaging databases are needed when the imaging appearance of patients with a certain condition is compared to that of healthy controls without the condition, and to develop new methodology [1]. Control databases are especially critical when it comes to statistical analysis [2, 3] including machine learning [1].

There has been an increase in neuroimaging database availability over the years; however, databases mainly consist of MR (Magnetic Resonance) images rather than PET (Positron Emission Tomography), perhaps due to many countries having restrictions on exposing healthy controls to ionising radiation.

There are very few [^18^F]FDG-PET (Fluorodeoxyglucose PET) databases available publicly or on request. Archambaud et al. [4] described 24 children (mean ± SD 11 ± 3.1 years, range 5–18, transmission-based attenuation correction) who were “pseudo-normal”, i.e. no abnormality was found on visual or statistical analysis. Wei et al. described another database of 78 “pseudo-normal”, Western Chinese, participants (mean 45 years, range 3–78, PET-CT, filtered back projection reconstruction [5]) whose brain was included in the field of view when they had half-body [^18^F]FDG PET for cancers not involving the brain; the database was tested against a single patient with epilepsy. Waterschoot et al. 2021 described another database of 83 healthy subjects (18–76 years, point-source transmission attenuation correction, 3D row action maximum likelihood algorithm [6]). The Alzheimer’s Disease Neuroimaging Initiative (ADNI, http://adni.loni.usc.edu) has [^18^F]FDG PET images for over 300 healthy controls (e.g. *n* = 360 used in Ding et al. [7]) but is restricted to higher ages (e.g. ages ~ 76 ± 6 years, range 60–96, in Ding et al. [7]). Several commercial packages contain normal [^18^F]FDG brain PET data, but typically with scant information [6, 8]. We have recently provided a detailed description of MRXFDG, a collection of healthy controls specifically scanned for databasing, consisting of [^18^F]FDG brain PET-CT as well as CTs, T1 weighted, and FLAIR MR images (*n* = 37, 38 ± 11.5 years, range 23–65).

The brain PET databases listed above have major differences due to hardware used. Differences include spatial resolution and field of view; attenuation correction method; preparation; dose injected; length of uptake and scanning time; and reconstruction method. It has previously been shown that differences in the acquisition of PET data between groups reduces the accuracy of classifying dementia patients [8, 9], hence adequately harmonised databases also have the potential improve existing diagnostic techniques in dementia [9]. However, we were mainly interested in comparisons against younger patients or research participants with younger age ranges. As databases are particularly scarce for younger adults, their combination is—in principle—an attractive option but needs investigation.

Investigations into harmonizing [^18^F]FDG-PET scans that have been acquired using different scanners and/or protocols exist for phantom studies [10–12], and for harmonizing radiomics features in half-body PET [13]; however, few studies have investigated harmonisation of brain images obtained with [^18^F]FDG [9, 14] or other ligands [15]**.**

Clinically, combining databases of younger adults is particularly important for epilepsy, where the mean age at presurgical evaluation is typically around 30 years of age [16], and where FDG PET coregistered with MRI is crucial for finding small areas of focal cortical dysplasias (FCDs) [17–20].

This study investigates the combination of two [^18^F]FDG-PET and T1 weighted MRI neuroimage databases: CERMEP-iDB-MRXFDG, which was characterised in Merida et al. [1] and a database created in Marseille which underwent similar characterisation in this study. Varying combination strategies and statistical designs were used to compare detection rates for histologically verified FCDs [21–23] in the preoperative scans of 34 patients obtained on three different scanners, using the standard software statistical parametric mapping (SPM12, https://www.fil.ion.ucl.ac.uk/spm/software/spm12/).

## Materials and methods

### Cohort characteristics

#### Marseille database

The Marseille database consists of 60 healthy volunteers, but MRI was unavailable in two. The remaining *n* = 58 volunteers with complete datasets (34 women) had an age of 50 ± 17 (range 21–78) years.

Participants had given written informed consent. Full inclusion and exclusion criteria can be found at https://clinicaltrials.gov/ct2/show/NCT00484523. A 15-min acquisition of PET data started 30 min after injection of 111 MBq of [^18^F]FDG. Participants were instructed to rest with their eyes closed during the uptake period. PET was obtained on a GE Discovery ST PET/CT (GE Healthcare, Waukesha, Wisconsin, USA), with a 6.2 mm axial resolution. Images were CT-corrected for attenuation and reconstructed into 47 contiguous 3.27 mm thick transverse sections using the ordered subsets expectation maximisation algorithm (OSEM), with 5 iterations and 32 subsets [24]. The final images had a voxel size of 1.17 mm isotropic in a matrix of 256 × 256 × 256 voxels.

Sagittal T1-weighted anatomical images were acquired on a Siemens SymphonyTim 1.5 T scanner (voxel size 1 mm isotropic, TR 1880 ms, TE 2.92 ms, inversion time 1100 ms, flip angle 15°).

#### iDB-MRXFDG database

MRXFDG [1] is a database of anatomical MRI and PET [^18^F]FDG head images from 37 normal adult human subjects (age 38 ± 11.5 years, range 23–65). The PET data were acquired for 10 min on a Siemens Biograph mCT64 50 min after administration of a planned dose of 1.5 MBq/kg + 18.5 MBq of [^18^F]FDG (with an actual dose injected of 122.3 ± 21.3 MBq [1]). PET images were reconstructed with OP-OSEM 3D (12 iterations, 21 subsets), incorporating the system point spread function and time of flight in a matrix of 200 × 200x109 voxels with a voxel size of 2.04 × 2.04 × 2.03 mm^3^.

Sagittal T1-weighted MRI data were obtained on a Siemens Sonata 1.5 T scanner (voxel size 1.2 mm isotropic, TR 2400 ms, TE 3.55 ms, inversion time 1000 ms, flip angle 8°).

#### Patient group

[^18^F]FDG PET data for 34 patients (17 men, 27 ± 11.9 years, range 13–48) were available from an ongoing service evaluation (King’s College Hospital, London, project reference 10/2021). All had histopathologically proven FCD type 2 [21–23]. [^18^F]FDG uptake is regularly decreased in FCD type 2 [17–19], and detection of decreased uptake was therefore used as an outcome parameter in this study. Patients were scanned 1994–2017 on three different imaging systems: a GE Discovery 710 with a full-width-at-half-maximum NEMA resolution of 5.3 mm (“VPFX”, a time-of-flight iterative OSEM, Ordered Subsets Expectation Maximisation reconstruction method), a GE Discovery ST with a resolution of 6.4 mm (Iterative Reconstruction (IR) or filtered back projection (FBP)) and a CTI ECAT 951/R with an estimated resolution on the image of ~ 8.9 mm [25] (FBP).

### Image processing

We used SPM12 (https://www.fil.ion.ucl.ac.uk/spm/software/spm12/) and SPM8 (https://www.fil.ion.ucl.ac.uk/spm/software/spm8/), implemented in MATLAB.

Control data had been anonymised before transfer. The patient Digital Imaging and Communication in Medicine (DICOM) files were anonymised and converted with *dcm2niix* (MRIconGL software) to NIFTI format for SPM analysis.

#### Processing of the Marseille database

##### Visual inspection

Images had already been reviewed by the creators of the database, EJ (Consultant Nuclear Medicine physician) and NG (Consultant Neuroradiologist). For the purposes of this analysis, images were additionally visually inspected by SOJ (Master student) and AH (Consultant Neurologist). Subject 1 was excluded from further analysis due to an incidental finding of a medial frontal arachnoid cyst which would have led to exclusion of this area in all subjects in SPM.

##### SPM analysis (Fig. 1)

**Fig. 1:**
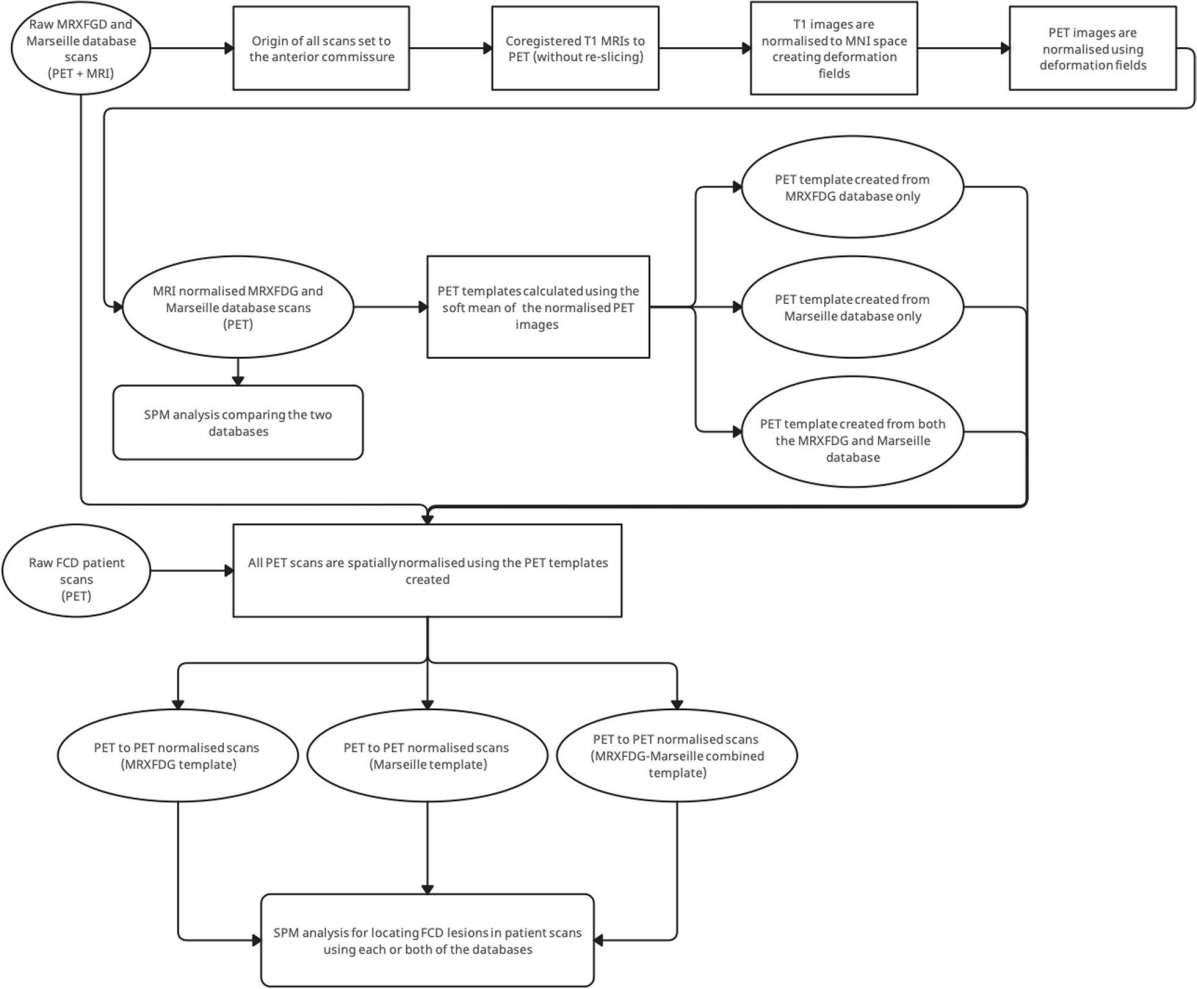
Flowchart of SPM analyses comparing the MRXFDG and Marseille databases and comparing individual FCD patients against separate and combined databases. For details, see text

##### Reorientation and coregistration

The origin of each PET and MRI image was manually set to centre of the images, and images were manually straightened using the pitch, yaw and roll reorient options in SPM. The origin was set approximately to the anterior commissure. All images were then coarsely aligned (but not resliced). Each T1-weighted MR image was finally coregistered (without re-slicing) to its corresponding PET image.

##### Spatial normalisation

Coregistered T1 images were normalised to MNI space with the *Segment* function yielding a deformation field for each participant. PET images were normalised with the *Normalise and Write* function using those deformation fields and resampled at 1 × 1 × 1 mm voxel size using a 4th degree B-spline interpolation.

##### Intensity normalisation

Reconstructed PET images were normalised by (1) each subject’s weight and injected dose to obtain Standard Uptake Value (SUV) images (radioactivity concentration [kBq/cm^3^]/(decay-corrected dose [kBq]/weight [kg])) and (2) by each subject’s mean activity within the intracranial volume (ICV) mask provided by SPM12 to obtain Standard Uptake Value ratio (SUVr) images.

##### Leave-one-out SPM analysis

Each of the participants was compared to the others via leave-one-out ANCOVA with images smoothed using an 8 × 8 × 8 mm full-width-at-half-maximum (FWHM) 3D Gaussian filter. Global values (calculated by SPM via its default method of taking the overall image matrix mean, taking 1/8th of it leading to a brain mask, and averaging within this mask) and age were used as linear covariates. Other settings were: grand mean scaling and ANCOVA; independent measurements; equal variance; overall mean for centring; relative threshold masking (0.8); implicit but no explicit masking; global calculation omitted. Global normalisation with overall grand mean scaling (to 100), ANCOVA. Hypometabolism (reduced uptake in the individual compared to the group) and hypermetabolism contrasts were investigated. Clusters were formed by voxels exceeding the threshold of *p* < 0.001 and were considered significant if their *p* value was < 0.05 at the cluster level (i.e., multiple comparison corrected via random field theory).

##### Regional analysis

The T1 MR images were anatomically segmented into 95 regions of interest (ROI) of the Hammers atlas database (http://brain-development.org/brain-atlases/adult-brain-atlases/individual-adult-brain-atlases-new/) [26–29] using the multi-atlas propagation with enhanced registration (MAPER) method [30]. Grey and white matter parts of mixed ROIs were separated using the approach described in Merida et al. [1]. Mean regional SUV and SUVr of the MR-coregistered PET were extracted in a selection of grey matter anatomical regions. We left out the white matter region corpus callosum and the ventricles, and combined all insular subregions into one label for comparison with Mérida et al. 2021. Right and left regions were combined.

The intra-regional coefficient of variation (COV) was calculated per participant and anatomical structure as the ratio between the SUV standard deviation and the mean SUV to represent regional SUV heterogeneity.

#### Processing of MRXFDG database

This had followed the same principles as for the Marseille database. Briefly, the images had been reviewed by two neurologists; the database included CT, T1, MRI, and FLAIR MRI and were coregistered to the PET scans using SPM12. Spatial normalisation was done in SPM12; intensity normalisation was conducted by calculating SUV (using subjects’ weight and injected dose) and SUVr (using the mean ICV mask provided by SPM12). Leave-one-out analyses had been conducted using the same methods used for the Marseille database. Region definition had been conducted using the same Hammers Atlas Database (i.e., the same regions) as for the Marseille database, but with the simpler method whereby a single maximum probability map in MNI space (containing information from all 30 atlases) was transferred back to individual space via the deformation fields derived from SPM (i.e. one registration per participant as opposed to 30 independent registrations per participant for MAPER). Full details of the process are provided in Merida et al. [1].

#### Processing of patient group

The patient data were manually reoriented and realigned as above. However, as no MR images were available, normalisation to MNI space was performed using PET templates (cf. Figure 1). Three different templates were created from the PET images of control subjects normalised via their corresponding T1 MRI: (1) with the Marseille database only, (2) with iDB-MRXFDG only and (3) with both databases, each time using a ‘soft mean’ [31] equation within SPM’s *imcalc* function:

$${\text{Soft}}\;{\text{mean}} = \frac{{\sum\nolimits_{n = 1}^{N} {i_{n} } }}{{\sum\nolimits_{n = 1}^{N} {\left( {i_{n} \sim = 0} \right)} }}$$

where *N* is the total number of subjects and *I* is each PET image in the database.

The *Normalise Estimate and Write* function in SPM8 was then used to normalise the patient PET images to each of the three templates, resulting in three sets of normalised images. This PET-to-PET normalisation was then performed for both databases using their respective templates for comparison with patient data to ensure the database-specific normalisation method (i.e. MRXFDG controls normalised to the MRXFDG-derived template were compared with patients normalised to the MRXFDG template; Marseille controls normalised to the Marseille-derived template were compared with patients normalised to the Marseille template; combined controls normalised to the combined template were compared with patients normalised to the combined template; cf. Figure 1).

### Merging of control databases

For the Marseille and MRXFDG databases, we evaluated: (1) compatibility of resolution level, (2) systematic SUV differences at the voxel level, (3) age correlation with SUVs.

#### Image resolution

From visual inspection and as expected, the Marseille database had a lower resolution than MRXFDG. Such differences may be compensated by smoothing to a common resolution [14].

**Fig. 2:**
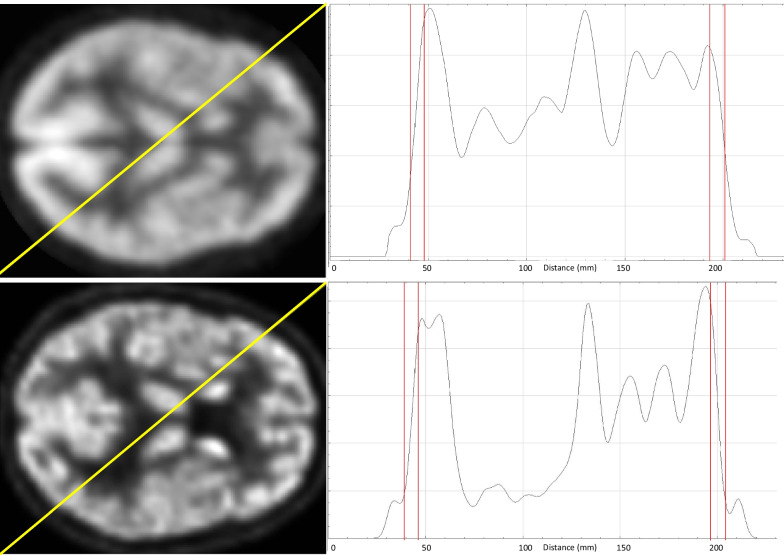
Method for determining smoothness: selected slice and profile line (left side of Figure) and corresponding intensity profile (right side of Figure) for one subject of the Marseille database (top row) and one subject of iDB-MRXFDG (bottom row)

To determine the amount of smoothing needed for MRXFDG to reduce differences prior to SPM analysis, normalised PET images were smoothed with a Gaussian kernel of 8 × 8 × 8 mm FWHM, and MRXFDG additionally with a 10 × 10 × 10 mm FWHM kernel. We then selected an axial slice in the middle of the brain (slice 78) from 15 normalised ± smoothed PET images in each database, extracted intensity profiles from a diagonal line (Fig. 2), and measured the mean outer cortical gradient on the intensity profiles (between pixels 40–50 and 195–210). Absolute values of the gradients were compared and differences assessed via two-sample t tests with equal variance.

The optimised smoothing levels derived from this analysis were used in all subsequent experiments.

#### Age correlation analysis

Total brain volume decreases nonlinearly especially beyond the age of 60 [2, 32]. Correlations between [^18^F]FDG uptake and age were assessed with SPM12 on each control database. We then removed the oldest subjects by decade of age and repeated the analyses until most correlations disappeared.

#### Voxel-based differences in [^18^F]FDG maps

The control databases were compared voxel-wise with a two-sample t test in SPM using the smoothing levels derived from the analysis of smoothness described above. The global values were taken into account as described above (section “Processing of the Marseille database—Leave-one-out SPM analysis”) including age as a linear covariate. Other settings were as above. Clusters were again formed and corrected for multiple comparisons as described above.

### Detection of FCD-related decreases in [^18^F]FDG uptake

To assess the FCD-related decreases in [^18^F]FDG uptake in patients with FCD in comparison with healthy subjects, different SPM models and ways to combine the two databases were explored, using the database-appropriate template as described above in the “Processing of patient group” section.

#### Analysis against each control database separately

An ANCOVA with group factor and age as covariate was performed against each database independently with the following settings: independent measurements; equal variance; grand mean scaling and ANCOVA; overall mean for centring; relative threshold masking (0.8); implicit but no explicit masking; global calculation omitted. Global normalisation with overall grand mean scaling (to 100), ANCOVA.

#### Analysis against combined control databases

##### Two-sample *t* test

A two-sample *t* test with age as covariate was conducted as for the separate control groups, simply combining the two databases into one.

##### Full factorial design

The full factorial design had three levels (patients and the two control groups) assumed to be independent (i.e., the global intensity ANCOVA was performed per group) and age as covariate. The other settings were the same as for the two-sample *t* tests.

##### Statistical assessment of patient results

We defined correct detection of the FCD as an area of significant decrease in uptake at the location of the area of resection of the histologically verified FCD.

Significant clusters were defined as in the leave-one-out analysis. However, as sensitivity is more important than specificity when screening for potential targets for intracranial EEG exploration, when no clusters were found at the initial threshold of *p* < 0.001, we also interrogated the SPMs at *p* < 0.01.

#### Influence of scanner type and patient characteristics

We examined the detection rate for patients scanned on the three different cameras, using the best model derived above.

We also compared detection rates for patients whose FCD had been detected on MRI versus those who had been “MRI-negative”. The MRIs were classified as negative or positive after visual analysis by a neuroradiologist who had clinical information available both during reporting and during the multidisciplinary epilepsy surgery meetings.

## Results

### Marseille database

#### Leave-one-out SPM analysis

Of the 58 healthy volunteers with PET and MRI in the Marseille database, two (#20, 55) had extensive areas of hypermetabolism (increased [^18^F]FDG uptake) and one (#6) had extensive areas of hypometabolism (decreased [^18^F]FDG uptake). A fourth subject (#1) had an arachnoid cyst in the medial frontal lobe which would have led to masking out of this area in all SPMs (see visual analysis above). These four control subjects were excluded from all further analyses.

Results for the initial (*n* = 58) and final (*n* = 54) group are listed in Table 1.

**Table 1:** Results of the leave-one-out analysis: false positives

Contrast	Subject-level (%)	Cluster-level (%)	Voxel-level (%)
*N* = 58 *subjects*			
Hypermetabolism	12.1 (*n* = 7)	7.4	0.98
Hypometabolism	12.1 (*n* = 7)	7.4	1.1﻿0
*Removing the aforementioned subjects from this analysis* (*n* = 54)			
Hypermetabolism	8.6 (*n* = 5)	3.7	0.45
Hypometabolism	10.3 (*n* = 6)	4.2	0.41

At the subject-level, the denominator is the total number of participants; at the cluster-level, it is the average number of resolution elements in the mask; at the voxel-level, it is the number of voxels inside the SPM mask

#### Demographics

The Marseille database group of 60 participants had a mean age of 50 years (standard deviation 16.5, range 21–78). There were 35 females and 25 males. Average weight was 67 kg (± 13.3, range 45–103), average height was 166 cm (± 9.5, range 149–195). All subjects had an injected dose of 111 MBq. The full demographics table indicating the two participants without MRI and the four removed after leave-one-out analysis is shown in the Additional file 1.

#### Regional analysis

**Fig. 3:**
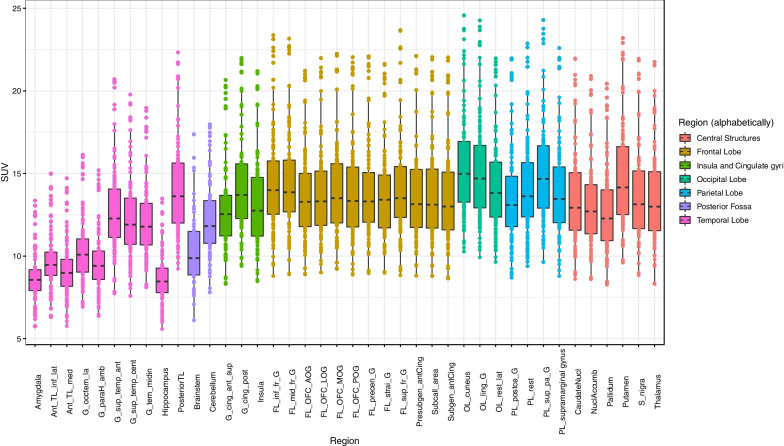
Regional SUV for all retained subjects (*n* = 54) in the Marseille database. Centre lines = medians, boxes = interquartile ranges, whiskers = robust ranges (1.5 × interquartile ranges). Outliers are represented as dots. Each dot represents a participant for unpaired regions and a participant’s right or left SUV value for paired regions. TL, temporal lobe; ant, anterior; inf, inferior; lat, lateral; med, medial; G_occtem_la, lateral occipitotemporal (fusiform) gyrus; G_paraH_amb, parahippocampal and ambient gyrus; G_sup_temp_ant, superior temporal gyrus, anterior part; G_sup_temp_cent, superior temporal gyrus, central part; G_tem_midin, middle and inferior temporal gyrus; G_cing_ant_sup, anterior (superior) cingulate gyrus; G_cing_post, posterior cingulate gyrus; FL, frontal lobe; FL_inf_fr_G, inferior frontal gyrus; F_mid_fr_G, middle frontal gyrus; OFC, orbitofrontal cortex; AOG, anterior orbital gyrus; LOG, lateral orbital gyrus; MOG, medial orbital gyrus; POG, posterior orbital gyrus; precen_G, precentral gyrus; strai_G, straight gyrus; sup_fr_G, superior frontal gyrus; Presubgen_antCing, presubgenual anterior cingulate gyrus; subcall_area; subcallosal area; subgen_antCing, subgenual anterior cingulate gyrus; OL, occipital lobe; ling_G, lingual gyrus; rest_lat, lateral remainder of occipital lobe; PL, parietal lobe; postce_G, postcentral gyrus; PL_rest, angular gyrus; sup_pa_G, superior parietal lobule; Nucl, nucleus; S_nigra, substantia nigra

Per-region mean absolute SUVs and the standard deviation of those means were 13.0 ± 1.8 (coefficient of variation 14%), with a range of individual values of 5.6–24.6. Absolute SUVs for the 54 participants grouped by lobe or brain area (Fig. 3) showed a relatively wide spread between participants, with similar distribution for most of the areas (median SUVs ~ 13–15 with ranges of ~ 8–25). However, temporal lobe and posterior fossa regions had slightly lower values and higher variability, with median SUVs ~ 10–11 and ranges of ~ 5–23.

**Fig. 4:**
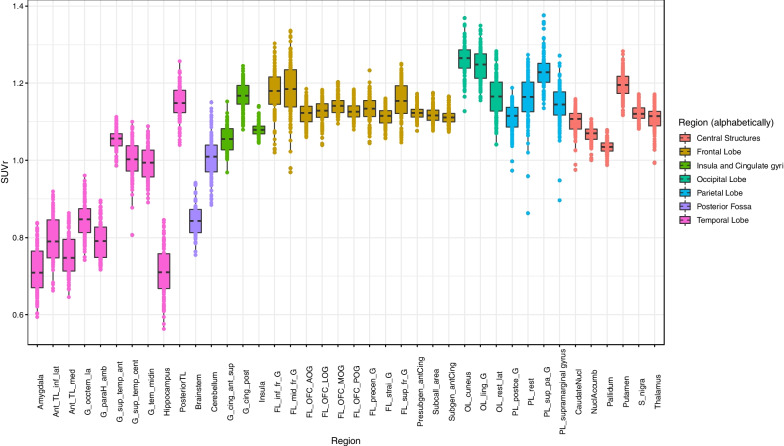
Regional SUVr obtained by normalizing by mean SUV in ICV mask for all subjects retained (*n* = 54) in the Marseille database. Graphics and abbreviations as in Fig. 3

After normalisation of SUVs by individual intracranial SUV to yield SUVrs, mean SUVrs and the standard deviation of those means were 1.1 ± 0.15 (coefficient of variation 14%), with a range of individual values of 0.56–1.38. SUVrs for the 54 participants grouped by lobe or brain area (Fig. 4) showed substantially reduced inter-subject variability compared with SUVs, and SUVrs also revealed inter-regional differences much more clearly.

### Database comparability for healthy controls

#### PET image resolution and smoothing level

Table 2 shows the gradients calculated to assess smoothness of the Marseille and iDB-MRXFDG databases for normalised ± smoothed images. When both databases were smoothed with the 8 mm Gaussian smoothing filter, the gradients of the smoothed images were significantly different (*p* < 0.05). Smoothing the Marseille database at 8 mm and iDB-MRXFDG at 10 mm led to no significant difference (*p* > 0.05). Smoothing also reduced the between-subject variance of the gradient as expected.

**Table 2:** Smoothness gradients

Gaussian filter used	Gradient (mean ± SD)	Coefficient of variation (%)
Marseille database		
No filter	21.4 ± 3.1	14.5
**8 × 8 × 8 mm**	**15.9 ± 1.5**	9.4
iDB-MRXFDG		
No filter	22.2 ± 4.0	18.0
8 × 8 × 8 mm	17.2 ± 2.5	14.5
**10 × 10 × 10 mm**	**15.0 ± 2.1**	14.0

A summary of the gradients calculated to assess to smoothness of the normalised and/or smoothed databases. Optimised smoothing level retained highlighted in bold

All following analyses used these optimised smoothing levels.

#### Regional SUVs

**Fig. 5:**
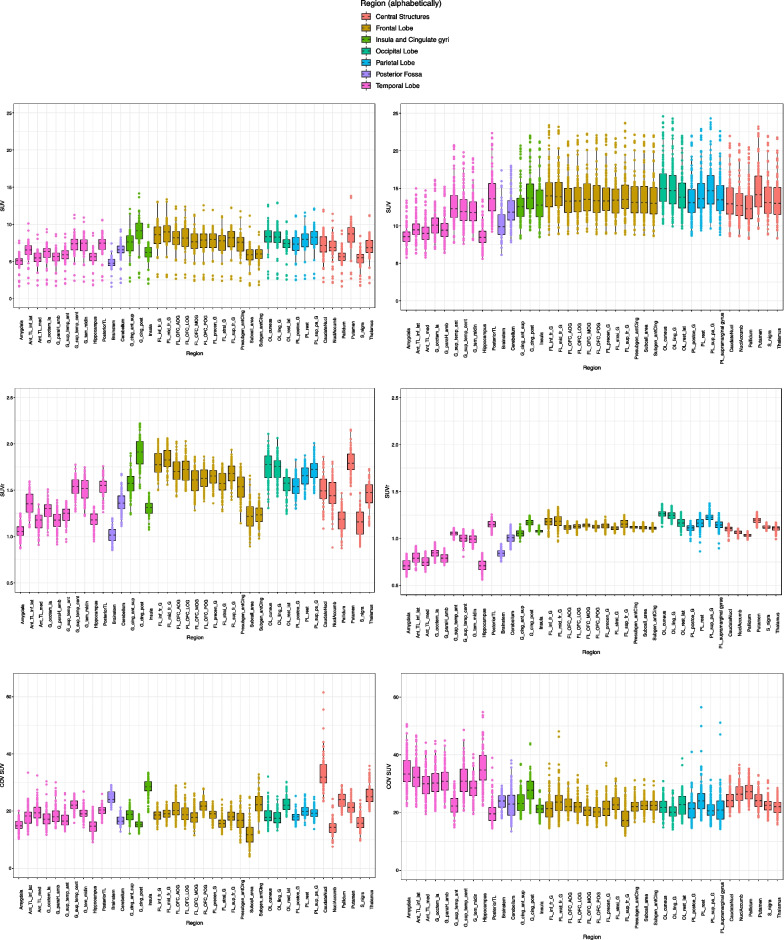
Regional SUV (row 1), SUVr (row 2) and SUV coefficient of variation (row 3) for MRXFDG (left column) and Marseile database (right column). Abbreviations as in Fig. 3

Regional SUVs had similar patterns across cerebral regions between MRXFDG and the Marseille database (Fig. 5, row 1). Overall, higher SUVs were observed in the Marseille database (regional median ~ 10–15, against ~ 5–10 for MRXFDG), as well as a higher dispersion in SUVs across subjects (note the fixed-dose injection compared with weight-adjusted injection for MRXFDG). In contrast, a higher inter-regional variability was observed in MRXFDG, in line with MRXFDG’s higher spatial resolution.

SUVs normalised by individual intracranial volume (SUVr) were higher for MRXFDG (median SUVr ~ 1–2 vs. ~ 0.7–1.2 for the Marseille database, Fig. 5, row 2), again in line with the higher spatial resolution in MRXFDG. Higher between-subject SUVr variability was also observed for MRXFDG. In addition, MRXFDG also had higher inter-region SUVr variability, in particular when comparing regions of the frontal lobe and central structures between databases.

Intra-regional COVs (Fig. 5, row 3) were similar for both databases, except for caudate nucleus (higher values and dispersion in MRXFDG) and temporal lobe regions (higher values and dispersion in the Marseille database).

#### Age correlation

##### Marseille database

For the 54 subjects, age was positively correlated with [^18^F]FDG uptake in five white matter clusters (all *p* < 0.05): right and left paracentral hemisphere, cerebellar white matter encompassing left and right, and left and right posterior crus of the internal capsule and temporal stem. There was a negative correlation with [^18^F]FDG uptake in two clusters (both *p* < 0.005), encompassing frontal poles, medial frontal lobes and anterior cingulate gyri, lateral frontal lobes extending into the Sylvian fissures, and caudate nuclei.

When volunteers aged 60 years or older were removed (reducing the database to *n* = 35), there were no significant increases with age and only two clusters of decreased [^18^F]FDG uptake with age along fissures, in the anterior interhemispheric fissure and right Sylvian fissure/insula (*p* < 0.05).

##### iDB-MRXFDG

Age across all 37 scans was positively correlated with [^18^F]FDG uptake in two white matter clusters (both *p* < 0.05), in posterior pons extending into the left crus cerebri, and right crus cerebri; uptake was negatively correlated with [^18^F]FDG uptake in two clusters (both *p* < 0.05), in the right anterior superior frontal gyrus near the superior frontal sulcus, and the interhemispheric fissure near the anterior cingulate sulcus.

For the *n* = 35 scans from volunteers under 60 years old, there was a single borderline significant cluster of positive correlation between age and [^18^F]FDG uptake in the right superior medial occipital lobe (*p* < 0.044) and a single cluster of negative correlation centred on the superior frontal interhemispheric fissure (*p* < 0.005).

#### Voxel-wise two-sample t test between databases of healthy controls

In the two-sample t test with age as a covariate, comparing 54 scans from the Marseille database with the 37 scans from iDB-MRXFDG, in the Marseille relative to the MRXFDG database there were two areas of significantly increased uptake (*p* < 0.05) in the left and right occipital poles. As the global values in the two databases were very different and the two-sample t test does not correct for this, there was one very large significant cluster of decreased uptake (*p* < 0.001) encompassing most of the brain with a fronto-temporal emphasis but sparing the occipital lobe.

For the *n* = 35 participants under the age of 60 from each database, similar results were obtained.

#### Detection of FCD-related hypometabolism

##### FCD detection as a function of statistical model

As shown in Table 3, around half of FCDs were detected with either database. The simple combination of both databases led to worse performance despite adjusted smoothing levels. However, harmonising global values via the Full Factorial model improved performance, with the age-restricted combined databases performing best (up to 24/34 = 71% of lesions detected).

**Table 3:** Detection of FCD-related decreased [^18^F]FDG uptake in patients

	Marseille	MRXFDG	Both DB (2STT)	Both DB (FF—under 60 years)	Both DB (FF—all ages, linear age covariate)	Both DB (FF—all ages, quadratic age covariate)
Datasets in database(s) used	54	37	91	70	91	91
Lesions found (any *p* value)						
*n*	17	18	14	**24**	22	19
%	50	53	41	**71**	65	56
Lesions found (significant *p* value)						
*n*	14	18	12	**22**	19	18
%	41	53	41	**65**	56	53

Summary of detection of FCD-related decreased uptake in *n* = 34 patients, using any initial threshold (see text for details)

DB = database, 2STT = two-sided t test, FF = full factorial model

Best performance highlighted in bold

Initial voxel thresholds used were *p* < 0.001 and *p* < 0.01

Clusters were considered significant when their cluster *p* value was < 0.05

##### FCD detection as a function of PET scanner used

Using the best-performing model (Full Factorial, both databases, age restriction, any *p* value, see Table 3), detection rates were similar across patient scans obtained with the three scanners: 8/10 (80%) for the CTI ECAT 951/R, 9/14 (64%) for the GE Discovery ST, 7/10 (70%) for the GE Discovery 710.

##### FCD detection in MRI-negative cases

Eleven of the 34 patient scans had been “MRI-negative”, i.e., the FCD had not been detected on MRI. The location of the FCD was correctly identified in 10/11 (91%).

## Discussion

We characterised the Marseille database of [^18^F]FDG PET-CT and T1 MRI and then compared it to the recently described CERMEP iDB-MRXFDG database [1]. We investigated the harmonisation of both databases in the context of the example application of detection of epileptogenic lesions in patients with FCD.

### Marseille database

The Marseille database has previously been used in various contexts [24, 33] but has not undergone detailed characterisation as CERMEP iDB-MRXFDG [1].

In contrast to structural MRI, there is a dearth of databases of healthy controls with [^18^F]FDG-PET images published or made available to the scientific community (see introduction), especially in the younger age range which is essential for research into the epilepsies [18], disorders of consciousness [34, 35], and normal brain function [36–38]. The Marseille database meets this need since it is composed of subjects ranging from 21 to 78 years old (50 ± 17 mean ± SD) and can be used in a large panel of applications. Leave-one-out SPM analysis was conducted to ensure the normality within the database, resulting in 4 subjects (7%) being removed from further analyses, in line with Waterschoot et al. [6] where 4% of subjects were removed via a similar process.

### Database comparison

While overall the two databases were comparable in terms of [^18^F]FDG uptake patterns, we also found differences due to various factors.

First, global image appearance was different, with greater spatial resolution and better contrast in MRXFDG (Fig. 2). We demonstrated that this was due to different smoothness of the reconstructed images, expected from differences in acquisition systems and reconstruction algorithms. Reducing the differences in the image resolution (visually and quantitatively, see Table 2) was successfully achieved by smoothing the MRXFDG database with a larger Gaussian kernel, similarly to previous work [13, 14].

Second, different SUVs were obtained for the two databases (Fig. 5, row 1). As SUV is semi-quantitative, differences are expected between scanners and acquisition protocols, in particular for uptake delay and acquisition duration. For the Marseille database, the same dose was injected for all participants, which will explain the higher inter-subject variability compared to adjusting dose by weight, as in MRXFDG [1]. We also observed a deviation of the boxplots towards high SUV values for the Marseille database, especially in the occipital lobe, likely corresponding to more visual stimulation during the tracer uptake period. This was also borne out by the voxel-based comparison and occurred despite similar instructions, namely to rest with eyes closed. Finally, for MRXFDG, we observed three outliers with lower regional SUVs. They also had reduced inter-region variability suggesting they had not followed the fasting instruction, making the PET data less informative. Normalizing within-image via SUVr (Fig. 5, row 2) reduced between-subject SUVr variability for the Marseille database, as variability due to fixed-dose injection was compensated for. The MRXFDG database had higher inter-region SUVr variability, in particular in regions of the frontal lobe and central structures. This could be a consequence of the longer uptake period for MRXFDG (50 min vs. 30 min for Marseille) and the higher spatial resolution for MRXFDG which will enhance contrast between regions.

While intra-regional COV distribution was similar for the two databases (Fig. 5, row 3), we note: (1) intra-regional COV for the caudate nucleus in MRXFDG was higher than for all other regions, with a number of outliers. This may be due to the regional segmentation of MRXFDG via a single registration of a maximum probability atlas derived from the Hammers Atlas Database [26, 27] to the subject space which can result in caudate nucleus labels including voxels of the surrounding lateral ventricles, whereas the Marseille database was segmented via the 30 individual atlases from the Hammers Atlas Database (i.e., identical region definitions) but with the more accurate multi-atlas approach MAPER [30]. (2) Temporal lobe regions had higher intra-regional COV, higher variability, and a larger number of outliers for the Marseille database. This may be due to the shorter field of view of the scanner used for the Marseille database (15.7 cm vs. 22 cm for MRXFDG) which may produce higher COV in the temporal lobe which is closer to the edge of the field of view [39].

Third, correlations between age and [^18^F]FDG uptake were shown in each database, with positive correlations in the white matter (likely due to SPM normalizing to the global mean) and negative correlations in or near fissures widening with age. Leaving out subjects ≥ 60 years old minimised these in each database.

We used individualised smoothing of the two databases, based on the images themselves, ahead of SPM analysis. Another approach is to use standardised phantoms to derive a smoothing factor [40] to account for different reconstruction algorithms, later expanded to additionally account for different hardware (EQ.PET, https://marketing.webassets.siemens-healthineers.com/1800000002580275/475f0ba8d270/EQ-PET_WP_1800000002580275.pdf). This phantom-derived approach has also been applied for comparing half-body (i.e., non-brain) images across different reconstructions and PET systems [41]. However, as these methods only work on spatial resolution via Gaussian smoothing, brain PET with its strongly varying SUVs would still require normalisation for global uptake, as performed via a full factorial design in this study.

### Detection of epileptogenic regions in patients with FCD

We used the insights gained by the database analyses to explore combination of databases in an experiment designed to detect known abnormalities (FCD) (Table 3). Likely due to the global intensity differences, simply combining both databases decreased performance compared to each database on its own despite the much larger number of scans. Accounting for differences in intensity between the two databases in a full factorial design outperformed either database used separately. This was true for any type of accounting for age (via linear or quadratic covariates or by removing participants over 60 years old). However, excluding the oldest subjects in the databases informed by the age correlation experiments yielded the best results in locating FCDs (up to 71%); when all control participants were used, using age as a linear covariate was preferable to a quadratic fit but remained inferior to the exclusion of older controls.

Other studies have detected histologically proven MRI-negative FCD on PET SPM5 analysis against 30 controls in 72% [17], in line with our study. Detection rates on visual analysis were 78% for PET-only and 95% after coregistration with MR [17]. In another larger study from the same group, visual analysis blind to all electroclinical data led to a detection rate of 44% increasing to 71% with electroclinical data and to 83% after coregistration with MRI [18], suggesting the potential of using SPM or similar analyses of PET with larger databases jointly with MR data to increase detection rates in future studies [42].

### Limitations

Differences between databases could potentially be reduced by adapting the reconstructions to be more similar—for example, MRXFDG could have been re-reconstructed without the spatially varying point spread function. However, as PET hardware [43] and PET reconstructions [44] continue to evolve, such differences are unavoidable, and we opted to use both databases as provided. It is reassuring that we found similar performance for patient data acquired on three different systems, especially as we chose not to repeat the resolution matching for each of the three patient scanners which would be prohibitively time-consuming in practice.

We only investigated two [^18^F]FDG brain PET databases. As explained above, few are publicly available, and often the focus is on the older age group used for dementia studies. We found the best performance when restricting the age range, suggesting that e.g. the ADNI database exclusively containing data from ≥ 60 year-olds would be less suitable for applications like epilepsy workup or basic science studies in younger people.

We used a single sample of patient data as part of a Service Evaluation. However, there was variability from using three scanners, and the sample had the advantage of a histological ground truth.

### Perspectives

Our work contributes to the understanding of data structure and descriptive statistics of [^18^F]FDG PET databases. These have been studied less than MR databases but are crucial for detectability of pathology as shown here. Fully characterising [^18^F]FDG databases enables principled choices for combining them. In our work we have explored some harmonisation methods and shown a substantial improvement in the detectability of lesions, using FCD as an example. Future work can now focus on combining multiple databases and perfecting methods to decrease differences between databases, e.g. regional normalisation. In addition, while SPM is a well-understood analytical method, our work can be used beyond mass univariate testing towards combining databases in machine learning or artificial intelligence (AI) applications. As an example, we have recently created large databases of simulated healthy and FCD-typical brain FDG PET images from MR phantoms [45] and used them to create higher-quality PET images from clinical standard-resolution PET via convolutional neuronal networks, resulting in better quality metrics and FCD detectability. Healthy control databases and AI methods can also be used to simulate pseudo-healthy FDG PET from patients’ MRIs which can then be subtracted from the real clinical PET to reveal lesions [42, 46]. Real databases such as the ones examined in this paper fulfill the need to take into account the physics of image acquisition and reconstruction and inform of descriptive image statistics encountered in practice.

## Conclusions

Few brain PET research databases are available, and databases in commercial software are often poorly described. Our work contributes to plugging the gap in database information. We also show the crucial importance of database and image characteristics for research and clinical tasks. Via relatively simple steps, we provide guidance on how databases can be harmonised, and provide evidence that this can lead to better abnormality detection.

### Supplementary Information

**Additional file 1**. **Supplementary Table 1.** Demographics for individual participants and the group.

## Data Availability

The Marseille database is available following reasonable requests from Professor Eric Guedj. The MRXFDG database is available as described in Mérida I et al. 2021.
