# Supplementary material for: Characterisation of a novel [18F]FDG brain PET database and combination with a second database for optimising detection of focal abnormalities, using focal cortical dysplasia as an example
Source: EJNMMI Res. 2023 Nov 15;13:98. doi: 10.1186/s13550-023-01023-z (PMC10645721; doi:10.1186/s13550-023-01023-z)
Supplement: Supplementary file 1 — Additional file 1. Supplementary Table 1. Demographics for individual participants and the group. [file 13550_2023_1023_MOESM1_ESM.docx]

**Characterisation of a novel [^18^F]FDG brain PET database and combination with a second database for optimising detection of focal abnormalities, using Focal Cortical Dysplasia as an example**

Sameer Omer Jin, Inés Mérida, Ioannis Stavropoulos, Robert D. C. Elwes, Tanya Lam, Eric Guedj, Nadine Girard, Nicolas Costes, Alexander Hammers

### Supplementary material

Supplementary Table 1: Demographics for individual participants and the group

Supplementary Table 1: Demographics table. * Subjects without MRI, ** subjects removed after leave-one-out analysis. All subjects had an injected dose of 111 MBq. F=female, M=male, SD=standard deviation, Min=minimum, Max=maximum.
